# *Leishmania infantum* Ecto-Nucleoside Triphosphate Diphosphohydrolase-2 is an Apyrase Involved in Macrophage Infection and Expressed in Infected Dogs

**DOI:** 10.1371/journal.pntd.0003309

**Published:** 2014-11-13

**Authors:** Raphael De Souza Vasconcellos, Christiane Mariotini-Moura, Rodrigo Saar Gomes, Tiago Donatelli Serafim, Rafaela de Cássia Firmino, Matheus Silva e Bastos, Felipe Freitas de Castro, Claudia Miranda de Oliveira, Lucas Borges-Pereira, Anna Cláudia Alves de Souza, Ronny Francisco de Souza, Gabriel Andres Tafur Gómez, Aimara da Costa Pinheiro, Talles Eduardo Ferreira Maciel, Abelardo Silva-Júnior, Gustavo Costa Bressan, Márcia Rogéria Almeida, Munira Muhammad Abdel Baqui, Luís Carlos Crocco Afonso, Juliana Lopes Rangel Fietto

**Affiliations:** 1 Departamento de Biologia Geral, Universidade Federal de Viçosa, Viçosa, Minas Gerais, Brazil; 2 Instituto Nacional de Biotecnologia Estrutural e Química Medicinal em Doenças Infecciosas (INBEQMeDI), São Carlos, São Paulo, Brazil; 3 Departamento de Ciências Biológicas – ICEB/, Universidade Federal de Ouro Preto, Ouro Preto, Minas Gerais, Brazil; 4 Departamento de Bioquímica e Biologia Molecular, Universidade Federal de Viçosa, Viçosa, Minas Gerais, Brazil; 5 Departamento de Veterinária, Universidade Federal de Viçosa, Viçosa, Minas Gerais, Brazil; 6 Centro de Zoonoses de Governador Valadares, Governador Valadares, Minas Gerais, Brazil; 7 Núcleo de Bioinformática, Universidade Federal de Viçosa, Viçosa, Minas Gerais, Brazil; 8 Departamento de Biologia Celular e Molecular e Bioagentes Patogênicos, Faculdade de Medicina de Ribeirão Preto, Universidade de São Paulo, Ribeirão Preto, São Paulo, Brazil; Lancaster University, United Kingdom

## Abstract

**Background:**

Visceral leishmaniasis is an important tropical disease, and *Leishmania infantum chagasi* (synonym of *Leishmania infantum*) is the main pathogenic agent of visceral leishmaniasis in the New World. Recently, ecto-nucleoside triphosphate diphosphohydrolases (E-NTPDases) were identified as enablers of infection and virulence factors in many pathogens. Two putative E-NTPDases (∼70 kDa and ∼45 kDa) have been found in the *L. infantum* genome. Here, we studied the ∼45 kDa E-NTPDase from *L. infantum chagasi* to describe its natural occurrence, biochemical characteristics and influence on macrophage infection.

**Methodology/Principal Findings:**

We used live *L. infantum chagasi* to demonstrate its natural ecto-nucleotidase activity. We then isolated, cloned and expressed recombinant rLicNTPDase-2 in bacterial system. The recombinant rLicNTPDase-2 hydrolyzed a wide variety of triphosphate and diphosphate nucleotides (GTP> GDP  =  UDP> ADP> UTP  =  ATP) in the presence of calcium or magnesium. In addition, rLicNTPDase-2 showed stable activity over a pH range of 6.0 to 9.0 and was partially inhibited by ARL67156 and suramin. Microscopic analyses revealed the presence of this protein on cell surfaces, vesicles, flagellae, flagellar pockets, kinetoplasts, mitochondria and nuclei. The blockade of E-NTPDases using antibodies and competition led to lower levels of parasite adhesion and infection of macrophages. Furthermore, immunohistochemistry showed the expression of E-NTPDases in amastigotes in the lymph nodes of naturally infected dogs from an area of endemic visceral leishmaniasis.

**Conclusions/Significance:**

In this work, we cloned, expressed and characterized the NTPDase-2 from *L. infantum chagasi* and demonstrated that it functions as a genuine enzyme from the E-NTPDase/CD39 family. We showed that E-NTPDases are present on the surface of promastigotes and in other intracellular locations. We showed, for the first time, the broad expression of LicNTPDases in naturally infected dogs. Additionally, the blockade of NTPDases led to lower levels of *in vitro* adhesion and infection, suggesting that these proteins are possible targets for rational drug design.

## Author Summary

Visceral leishmaniasis is a dangerous and important, but neglected, tropical disease that affects millions of people, mainly in underdeveloped and developing countries. Presently, there are no vaccines against Leishmaniasis, and the few drugs with which the disease is treated have low efficacy and high side effects. The pathogenic agent of this disease in the New World is *Leishmania infantum chagasi*. In this work, we studied a protein from this parasite named ENTPDase-2. We expressed it in a bacterial system, purified it and characterized it as a genuine nucleotidase of the ENTPDase family. This protein seems to be localized at the surface of the parasite and in other intracellular locations. ENTPDase seems to facilitate *in vitro* infection because its blockade leads to lower levels of infection of macrophages. In addition, the protein is found in naturally infected dogs. A previous study demonstrated that ENTPDase-2 from *L. infantum chagasi* is a good antigen for immunodiagnosis of canine visceral leishmaniasis. We have now studied this protein in greater depth and suggest that it may be a good target for drug development.

## Introduction

Visceral leishmaniasis (VL) is a disease that, if not treated, is usually fatal. It affects thousands of people per year worldwide. The majority of cases occur in poor areas of underdeveloped or developing countries. This disease is considered an emergent public health problem because it is spreading to large urban centers in the endemic areas and because immune-deficient AIDS patients are highly susceptible [1]. There are few drugs, most of them highly toxic, to treat VL. Thus, the development of new drugs and other strategies to block, control and prevent the disease are still needed [2], [3].

Several parasites, including *Leishmania*, are dependent on the purine salvage pathway to synthesize purine nucleotides, which are required for the synthesis of nucleic acid and other important biological molecules [4]. The ecto-nucleoside triphosphate diphosphohydrolases are suggested to participate in the purine salvage pathway through the hydrolysis of extracellular nucleoside tri and diphosphates, leading to the production of nucleoside monophosphates. The ecto-5′-nucleotidases can then convert the nucleoside monophosphates to nucleosides, which can be taken into the cells to be used in intracellular purine nucleotide synthesis [5], [6]. In general, enzymes from the purine salvage pathway are considered good targets for rational drug design for diseases associated with pathogens that depend on this pathway [7].

The role of E-NTPDases in infectivity, virulence or purine acquisition of the pathogenic protozoan parasites has been investigated in *Trypanosoma cruzi* [8], *Toxoplasma gondii* [9] and the species of *Leishmania* that causes tegumental leishmaniasis [6], [10]–[12]. These studies suggested that the E-NTPDases have key roles in parasite infections through the subversion of extracellular nucleotide signaling pathways, particularly those involving ATP and ADP. In mammals, E-NTPDases participate in the control of purinergic signaling [13], and parasites seem to have developed a similar system to control host responses associated with purinergic signaling.

A previous study from our group and sequences deposited in GenBank demonstrate the presence of members of this protein family in trypanosomatids, such as *T. cruzi* and *Leishmania major* [14].

In this work, we studied the E-NTPDases of *L. infantum chagasi*. To study the nucleotidase activity, we compared the natural ecto-nucleotidase activity of the live parasites with the recombinant E-NTPDase named rLicNTPDase-2 of *L. infantum chagasi*. In addition, we investigated the subcellular localization of this protein and evaluated its involvement in macrophage infection and its natural expression in tissues from dogs with canine visceral leishmaniasis. These approaches provided important steps towards the elucidation of the role of this protein in *Leishmania* infection and could open new fields for the rational design of new drugs or other biotechnological applications.

## Materials and Methods

### Bioinformatics and phylogenetic analyses

Initially, representatives of trypanosomatids ENTPDases from the GenBank database were assessed. These included the following accession numbers of selected sequences: *Leishmania major* gi68124641 and gi68125368; *L. infantum g*i146079011, gi134068433 (both from JPCM5 strain); *Leishmania braziliensis* gi134059793 and gi134060473; *T. cruzi* giAAS75599.1. These sequences were aligned with representatives of the mammalian ENTPDases using CLC Workbench version 6.9.1. The representatives of the mammalian ENTPDases were selected based on a previous publication [15]. The signal peptides were predicated using SignalP 3.0 and TMAP. The trees and alignments were built with CLC Workbench version 6.9.1.

The phylogenetic analyses of the full length coding regions of the Trypanosomatids ENTPDases (TpNTPDases) were conducted using a Blast search of the *L. infantum* TpNTPDase-1 and Tp-NTPDase-2 (gi146079011, gi134068433) using NCBI, TriTrypDB, KEGG, UniProtKB/Swiss-Prot and OrthoMCL data banks. The sequences were selected with a threshold of 1E-20 for the expectancy value and using the full-length coding region (containing the start methionine, the 5 ACRs and lengths compatible with the known reference enzymes). Redundancy was eliminated using an in-house script. As an out-group we used the *Toxoplasma gondii* NTPDase (Q27895.1). The sequences were aligned using the software MUSCLE v3.8.31 [16], and the alignments were inspected manually by using MEGA 5.2 [17]. The program ProtTest v2.4 [18] was utilized to estimate the best amino acid substitution model (JTT +I +G) [19]. The phylogenetic trees were constructed using the Bayesian inference (BI) method with the software MrBayes v3.1.2 [20] with two chains of a Markov Chain Monte Carlo algorithm (MCMC) and 10 million generations, sampling every 1000 generations. A stationary distribution, analyzed by Tracer software (http://tree.bio.ed.ac.uk/software/tracer/) was achieved after 1 million generations. Then, 10% of the trees were used to produce the consensus tree. The average standard deviations of the split frequencies were 0.003060. The trees were visualized and edited using FigTree v.1.4.0. At the same time, another phylogenetic analysis was performed using the Maximum Likelihood method (ML) based on the JTT substitution model using MEGA5 software. The initial tree(s) for the heuristic search were obtained by the Neighbor-Joining method to generate a matrix of pairwise distances estimated using the JTT +I +G substitution model. A discrete Gamma distribution was used to model the evolutionary rate differences among sites (4 categories (+G, parameter  = 3.2088)). Both trees (built by MrBayes and ML methods) showed the same topology. The final tree shown was based on 29 amino acid sequences from the MrBayes method with the addition of bootstrap analysis from ML method. The branch lengths are measured in the number of substitutions per site.

### Cloning of rLicNTPDases ectodomain

Clones containing the complete ORF of NTPDase-2 of *Leishmania infantum chagasi* M2682 (AFX98106.1) were produced previously [21]. Briefly, the signal peptide prediction performed using Signal-P [22] showed a probable cleavage site between amino acid residues G31 and F32. TMAP [23] showed a transmembrane segment between the amino acids L17 and L40. From these analyses, the amino acid residue region between L41 and E425 was classified as belonging to the ectodomain. For PCR-based cloning in the pET21b (+) vector (Novagen, Merck KGaA, Darmstadt, Alemanha), the following primers pairs were designed: 5′-agtagctagc **atgctgctctcccca**-3′ 'and 5′-agctcgag **ttccatcttgagcaggaa**-3′. The 5′ regions flanking the ectodomain (bold) for both primers. The primers have restriction sites for *Nhe*I and *Xho*I endonucleases (underlined). The PCR product and the vector were digested with the same enzymes and ligated using T4 DNA ligase [24]. *E. coli* DH5α cells were transformed with the recombinant plasmid by the method of chemical processing. The positive clones were identified by colony PCR, digestion and sequencing with the T7 promoter and terminator. rLicNTPDase-1 (the segment between the amino acid 28 and 677) was cloned in the same manner as Lic-NTPDase-2, using (FW 5′atacatatg **aacccgcttcagtcg** 3′ and RV 5′ atctcgag **ggtaagagagaggag** 3′) as the primers and the *Nde*I and *Xho*I enzymes.

### Recombinant rLicNTPDase-2 heterologous expression and purification

The recombinant plasmid was purified from the DH5α cells. The construct pET21b/NTPDase-2 was used to transform *E. coli* strain BL21 (DE-3) RIL for protein expression. The confirmed clones were tested for expression in SOC medium. The pre-inoculum was grown overnight in 5 mL of LB medium containing 50 µg/ml ampicillin. The culture was then transferred to 0.5 L of SOC medium, and the cells grew until the OD_600_ reached 0.6. The induction was triggered with 0.25 mM IPTG and carried out for one hour at 37°C. The induced culture was aliquoted and centrifuged. The pellets of 100 mL aliquots (approximately 0.9 g cells) were stored at −80°C until use. For lysis, 4 mL of lysis buffer (100 mM Tris pH 8.0, 300 mM NaCl) plus protease inhibitors (Aprotinin, Leupeptin and pepstatin at 1 µg/mL) and lysozyme (1 mg/mL) was added to the pellets. After 30 minutes on ice, the sample was sonicated for six cycles of 10 seconds at 20 Hz and centrifuged at 12500 g for 30 minutes. The pellets containing the inclusion bodies were resuspended in 20 mL of wash buffer (50 mM Tris pH 8.0, 500 mM NaCl, 2 M urea), followed by another centrifugation step at 12500 g. This step was repeated once more. The remaining pellet, which contained a high concentration of the protein of interest in insoluble inclusion bodies, was dissolved in denaturating buffer (50 mM Tris pH 8.0, 500 mM NaCl, 10 mM imidazole and 8 M urea) and further purified by nickel affinity chromatography on an FPLC Akta Purifier GE. The sample was eluted with buffer containing 50 mM Tris pH 8.0, 500 mM NaCl, and 300 mM imidazole. The eluate was diluted ten times (10x) in refolding buffer (100 mM Tris pH 8.0, 600 mM NaCl, 1 mM GSSG, 2 mM GSH and 33% glycerol) and stored at 4°C. The activity assays were performed 24 hours later.

### Determination of protein concentration

The protein concentration was determined using a microplate-based Bradford method [25]. BSA was used for the standard curve. The concentration was confirmed by separation of 1 µg of BSA and 1 µg of the isolated protein using 10% SDS-PAGE and comparing the intensity of the bands after staining with Coomassie blue.

### Recombinant rLicNTPDase-2 enzymatic activity

The enzyme activity assay was performed by a colorimetric method for the determination of inorganic phosphate (Pi) (malachite green method) [26]. The assay was performed in 50 mM Tris buffer pH 8.0, 50 mM HEPES pH 8.0, and 3 mM MgCl_2_ or CaCl_2_ (as indicated in the text), 116 mM NaCl, 5.4 mM KCl and 2.5 mM nucleotide. To determine the pH dependence, in addition to the reagents mentioned, the buffer also contained 50 mM MES. The pH was adjusted for each reaction. The reaction was initiated with 1 µg of protein and incubated for 30 minutes at 37°C until it was stopped with 0.1 M HCl. For the determination of the concentration of free orthophosphate, the colorimetric reagent (0.2% malachite green and 10% ammonium molybdate 1∶3, both dissolved in 4 M HCl) was added. The blank reaction contained all reagents except the protein, which was added after stopping the reaction. The experiments using inhibitors were performed with the following concentrations: 300 µM of ARL, 300 µM of gadolinium chloride, 100 µM suramin, 2 mM sodium azide, or 3 µM of ammonium molybdate. Each inhibitor was used in separate tests.

### Anti-rLicNTPDase-2 polyclonal antiserum production and purification

The purified recombinant rLicNTPDase-2 was used to produce a polyclonal antiserum by immunization of a young adult female rabbit, purchased from the Universidade Federal de Viçosa (UFV) rabbit warren. Prior to the immunization, a blood sample was obtained from the ear marginal vein (pre-immune serum). Three immunizations were performed by the intradermal route, using 200 µg/500 µL with intervals of 21 and 15 days. The first immunization included an equal volume (500 µL) of complete Freund's adjuvant (Sigma), and the two subsequent immunizations included incomplete adjuvant. The immune serum was recovered 7 days after the third immunization. The pre-immune serum and immune antiserum were evaluated by dot blotting analysis, which showed recognition of rLicNTPDase-2 up to 1∶160,000. Only the immune serum was able to recognize the recombinant rLicNTPDase-2. The total IgG was purified by the caprylic acid method [27].

### Parasite culture and growth

*L. infantum* promastigotes M2682 previously frozen in liquid nitrogen were thawed and seeded in Grace's medium (Sigma Cell Culture) at 25°C. The starter cultures consisted of 1×10^5^ *Leish*/mL in GRACE's medium (pH 6.5) supplemented with 10% inactivated fetal bovine serum, 100 U/mL penicillin and 2 mM L-glutamine. The parasites were grown at 25°C for five days until use. The slides were stained with the panoptic stain.

### Western blot and SDS-PAGE analyses

Western blot assay and SDS-PAGE were performed as previously described by Russel *et al* [28]. For the Western blot, to recognize the recombinant proteins in the recombinant expression and purification assays, we used the monoclonal antibody anti-hexa-histidine as the primary antibody (GE Healthcare) and, as secondary antibodies, either anti-rabbit-IgG conjugated with FITC Sigma or anti-rabbit-IgG conjugated with peroxidase. A solution of 0.3% BSA was used for blocking. Image acquisition was performed in Phosphoimage (Fujifilm) at 475 nm for FITC staining. The peroxidase activity was detected as chromogenic staining using DAB.

### Ecto-NTPDase activity in live parasites

*L. infantum chagasi* (M2682) were grown in Graces' medium. Parasites from the log phase were washed and suspended in reaction buffer. Ecto-NTPDase activities were measured according to the previous work using *T. cruzi* parasites [14]. Briefly, the hydrolysis of ATP, ADP, AMP, GTP, GDP, UTP and UDP was measured by incubation of live parasites for 1 h at 30°C in reaction buffer (50 mM Hepes-Tris, 116 mM NaCl, 5.4 mM KCl, 5.5 mM D-glucose, 5.0 mM MgCl_2_) in presence of 5 mM nucleotide. The reaction was stopped by adding 0.1 M ice-cold HCl and the suspensions were centrifuged. Inorganic phosphate (Pi) was measured in aliquots of the supernatant using the malachite green method [26].

### Adhesion assays and infection of J774 macrophages

J774 macrophages were grown in CTCMC medium at 37°C, 5% CO_2._ The cells were seeded in 24-well plates, 1 mL containing 1.0×10^6^ cells per well, and were allowed to adhere for 90–120 min. The medium was removed, and the wells were washed twice with sterile PBS to remove the non-adherent cells. To perform the infection, we used 3−5×10^6^ parasites/mL (3 to 5 parasites per macrophage) for 30 minutes (adhesion) or for 3 hours (infection). The parasites were suspended in CTCM containing 10% FBS, then the wells were washed twice with sterile PBS to remove free parasites. The cultures were maintained at 37°C and 5% CO_2_.

### Immunolocalization of LicNTPDases in epimastigotes by confocal laser scanning microscopy

Immunolocalization in promastigotes during exponential phase growth was performed by confocal laser scanning microscopy, by methods similar to those previously described [29], [30]. The live parasites were washed twice in PBS and allowed to settle onto glass slides coated with 1% poly-lysine. After one wash with PBS, parasites were fixed with PBS containing 4% paraformaldehyde for 10 min. The samples were washed with cold PBS and blocked with 2% BSA/PBS for 30 min following incubation with the purified polyclonal antisera against rLicNTPDase-2 (dilution 1∶50) in 2% BSA/PBS for 1 hour at room temperature. The slides were washed in blocking solution and subsequently incubated for 30 min at 37°C with Alexa 488-conjugated goat anti-rabbit IgG secondary antibody (Invitrogen Life Technologies) at a dilution of 1∶4000. The glass slides were mounted with Prolong Gold Antifade Reagent (Molecular Probes) and examined by confocal microscopy (Leica, SP5) at the Faculdade de Medicina de Ribeirao Preto-USP, Ribeirao Preto, SP.

### Ultrastructural immunocytochemistry

For transmission electron microscopy analysis, log phase promastigotes were fixed in 4% paraformaldehyde, 1% glutaraldehyde, 5 mM calcium chloride, and 3.7% sucrose in a 100 mM sodium cacodylate buffer (pH 7.2). The samples were gradually dehydrated in alcohol at low temperatures, infiltrated, and finally embedded in LR White resin at 60°C. Ultrathin sections were collected on nickel grids of 300 mesh and incubated for 20 min at room temperature in 50 mM ammonium chloride in PBS at pH 7.2. Next, the sections were incubated in PBS at pH 8.0 containing 1.5% albumin and 0.01% Tween 20 for 20 min at room temperature and then overnight in the presence of purified anti-rLicNTPDase-2 (1∶400), this antibody was omitted from the control grids. The grids were washed in PBS and finally incubated with a 1∶30 dilution of a secondary 10 nm gold-conjugated goat anti-rabbit IgG for 60 min. The ultrathin sections were contrasted with solutions of 3% uranyl acetate and 0.2% lead citrate. All of the samples were observed and photographed in a transmission electron microscope (Zeiss EM 109) at the Núcleo de Microscopia e Microanálise at Universidade Federal de Viçosa, Minas Gerais, Brazil.

### Immunohistochemical assays

rLicNTPDase-2 was immunodetected in tissues from naturally infected dogs. Prescapular lymph nodes from 48 dogs previously diagnosed with canine visceral leishmaniasis were collected from Governador Valadares, Minas Gerais, Brazil, an endemic area of Brazil. All procedures and experimental animal protocols were conducted in accordance with Brazilian Society of Laboratory Animal Science (SBCAL/COBEA). The tissues were processed histologically and immunostained using an indirect immunoperoxidase technique. Specifically, the paraplast resin (SIGMA) from the 4.5 µM sections was removed by two passages in xylol for 30 minutes and then hydrated in decreasing alcoholic solutions (100%, 90%, 80% and 70%) for 5 minutes. The endogenous peroxidase was blocked with 3% hydrogen peroxide in methanol for 30 minutes at room temperature. The sections were washed with PBS pH 7.4 and the antigen recovery was carried out with 1 mg/mL trypsin in PBS pH 7.4 for 10 minutes at 37°C. To block the nonspecific binding sites, we incubated the slides with normal goat serum diluted 1∶10 in PBS pH 7.4 in a humid chamber for 45 minutes at room temperature. After this, rabbit IgG anti-rLicNTPDase-2 (1∶2000) was added for 24 hours at 4°C in the humid chamber. The sections were washed in PBS pH 7.4, and then, the secondary antibody, anti-rabbit IgG produced in Goat-HRP conjugate.(1∶10), was added. Finally, the sections were washed in PBS pH 7.4 for 10 minutes and placed immediately into the revealing solution (1 mg of diaminobenzidine, 1 µL of 30v H_2_O_2_ in 1 mL of PBS pH 7.4) for 5 minutes. After another wash, the sections were counterstained with Harris hematoxylin 1∶10 in PBS pH 7.4 for 20 seconds and subsequently dehydrated in alcohol, cleared in xylene and mounted with Entellan between the slide and coverslip. The resulting slides were mounted and observed using a Nikon ECLIPSE E6003 binocular optical microscope.

### Ethics statement

The Ministry of Health, through the Brazilian National Health Foundation (FUNASA) and, State and Local Health Departments, performs control of visceral leishmaniasis centered on the reduction of vector density and elimination of infected dogs. The dogs used in this study were from Brazillian program of control visceral leishmaniasis. Dogs were diagnosed with leishmaniasis and sacrificed as provided in the Decree no. 51,838, of March 14, 1963, and in the Resolution No. 1000, of May 11, 2012. According to Brazilian law in this specific situation there is no need for registration by an ethic committee in animal experimentation.

## Results/Discussion

### Sequence comparisons between trypanosomatides ENTPDases and mammalian E-NTPDases

**Figure 1:**
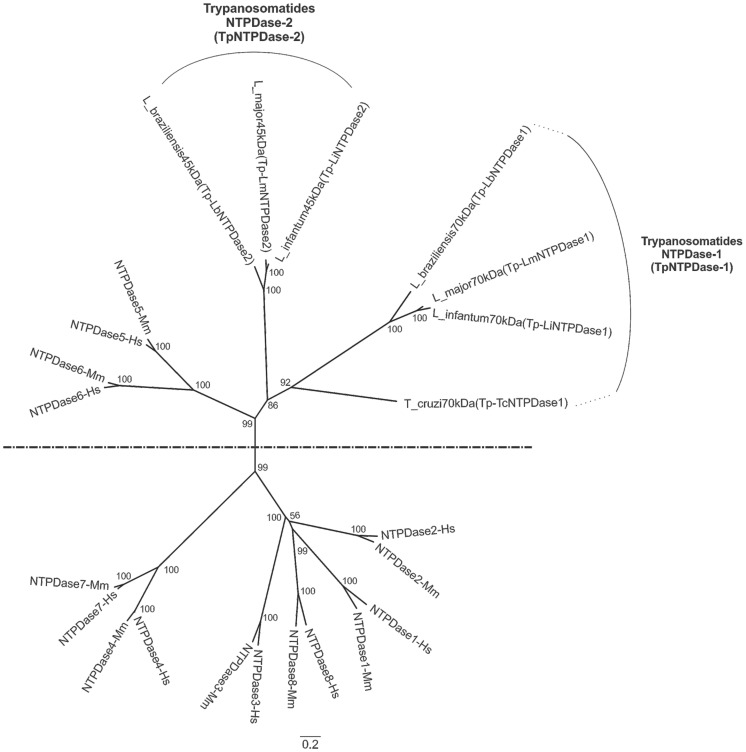
Phylogenetic tree using representatives of the ENTPDases from mammals and Trypanosomatids (*Leishmania* and *T. cruzi*). ENTPDase sequences were aligned by the CLC workbench program and used to construct the phylogenetic tree using the Neighbor Joining method with bootstrap analysis (number in the branches). *Mus musculus* (Mm); *Homo sapiens* (Hs). Trypanosomatids have two ENTPDases with exception of *T. cruzi*, which has only one ENTPDase in databank. Trypanosomatid ENTPDases are more similar to mammalian ENTPDases isoforms 5 and 6 and are grouped at the upper branch of the tree.

Mining the *L. infantum* genome (strain JPCM5), we found two putative isoforms of ENTPDases named as guanosine diphosphatase (gi 146079011) and nucleoside diphosphatase or ATP diphosphoydrolase (gi 146081775). These two putative proteins were aligned with those of *Leishmania*, *Trypanosoma cruzi* and the mammalian E-NTPDases representative of isoforms 1 to 8 from human and mouse (Figure S1). The mammalian ENTPDase representatives were selected according previous work [15]. As shown in figure 1, these ENTPDases form two distinct clades. One of them includes the mammalian ENTPDases 1, 2, 3, 4, 7 and 8 and the other clade includes mammalian ENTPDases 5 and 6 and ENTPDases representative of the trypanosomatids. Due to the higher similarity between the single *T. cruzi* ENTPDase-1 [14] and the *Leishmania* guanosine diphosphatase, we designated this group as trypanosomatids NTPDase-1 (TpNTPDase-1). All analyzed TpNTPDases-1 are predicted to be ∼70 kDa proteins and have an additional amino domain absent in other ENTPDases included in this work (Figure S1 and Figure S2). The other branch of the *Leishmania* NTPDases was named trypanosomatid NTPDase-2 (TpENTPDase-2) and includes the *Leishmania* proteins predicted to be ∼40 kDa (Figure 1).

Analyzing the amino acid identity between mammalian and trypanosomatid ENTPDases, it is clear that they are very divergent achieving the highest level of identity between *Mus musculus* ENTPDases-5 and *L. brazilienzis* TpNTPDases-2 (29.05%). In this analysis, we used the protein sequences of TpNTPDases-1 and TpNTPDases-2 from *Leishmania major*, *Leishmania infantum* and *Leishmania braziliensis*. The sequences were considered complete if they had approximately 425 amino acid residues for the TpNTPDases-2 or at approximately 670 amino acid residues for the TpNTPDases-1 and if they contained the initial methionine, the stop codon and Apyrase Conserved Regions (ACRs) 1–5, which are the amino acids involved in the catalytic site of these enzymes [29]. Considering the *Leishmania* and *Viannia* complexes [31], the TpNTPDases-2 exhibit ∼92% identity between species of the same complex (*L.(L.) major* and *L.(L.) infantum chagasi*) and approximately ∼77–79% identity between species from distinct complexes: e.g.*L.(L.) major* or *L.(L.) infantum chagasi* compared with *L.(V.) braziliensis*. The TpNTPDases-1 show ∼90% identity between enzymes from the same *Leishmania* complex and 67–69% between species from different complexes.

To expand the trypanosomatid ENTPDase analysis, we searched for TpNTPDases in the protein data banks. Initially, we found 82 TpNTPDases with *e-values* lower than 10^−20^, although after exclusion of redundant sequences, incomplete sequences or sequences without the 5 ACRs, only 29 sequences used in the next step of the phylogenetic analyses. Considering all amino acids in the primary sequences, we found 850 sequence elements, but only 354 were used in the construction of the tree because the others were found in positions with gaps. The trees were constructed using the Bayesian inference (BI) and Maximum Likelihood (ML) methods, and both resulted in the same topology. These results reinforce the precision of these data. As shown in Figure S2, the TpNTPDases formed two distinct clades: one of them, clade 1, was related to the TpNTPDases-1 (syn. guanosine diphosphatase at the genome annotation) and the other, clade 2, was related to the TpNTPDases-2 (syn. ATP diphosphohydrolases at the genome annotation). This clade separation presents strong statistical support with a posterior probability (PP) = 100 to BI and bootstrapping value (BV) = 100 to ML for clade 1 and posterior probability (PP) = 99 to BI and bootstrapping value (BV) = 98 to ML for clade 2. It is possible to note that each clade is formed from two groups: one from *Leishmania* genus and another from the Trypanosoma genus (statistical support PP = 100 and BV = 100). *T. cruzi* remains as the unique trypanosomatid that has only one TpNTPDase, the NTPDase-1 present in clade 1 (XP_809354.1, AAS75599.1 and EKF98171.1). This expansion of the TpNTPDase molecular analyses could contribute to future studies of these enzymes by the research community.

**Figure 2:**
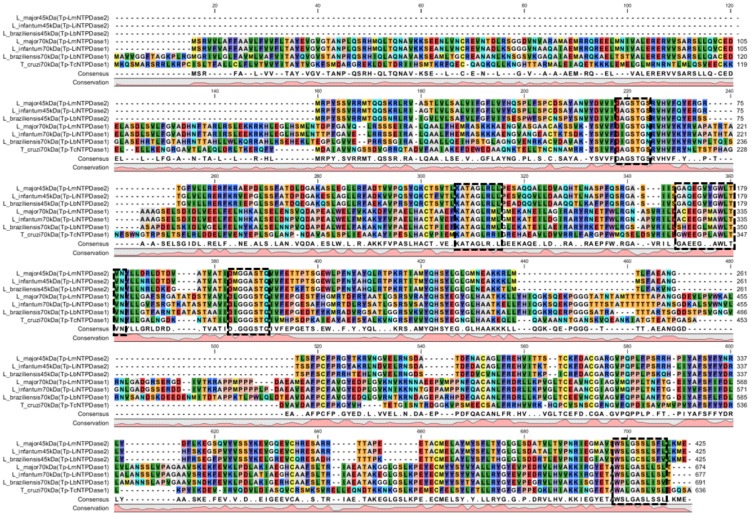
Alignment of representatives of TpNTPDases-1 and TpNTPDases-2. TpNTPDases from *L. infantum* (Li), *L. braziliensis* (Lb), *L. major* (Lm) and *T. cruzi* (Tc) were aligned using the CLC main workbench and manually inspected. The ACRs are shown in dashed line rectangles. The prevalent amino acids are shown in the consensus line and the level of conservation of each position at the primary sequences of proteins is shown in the conservation box.

It is noteworthy that there are differences between the ACRs of TpNTPDases-1 and TpNTPDases-2, even within one isoform in different species (Figure 2). We can hypothesize that these differences could be related to the biochemical differences observed among the ecto-nucleotidase activities of different *Leishmania* species because the ACRs are present in the catalytic sites of these enzymes [10]. It is important to emphasize the necessity to fully evaluate this hypothesis in the future.

### Cloning and expression of the soluble domain of rLicNTPDase

As the first step of characterization of the *L. infantum chagasi* NTPDase-2 (rLicNTPDase-2), we amplified and cloned the full coding region of LicNTPDase-2 from M2682 strain in the pGEM vector using the *L. infantum* JPCM5 strain (LiNTPDase) as a reference. Then, we sequenced the rLicNTPDase-2 gene and compared this sequence with the reference strain sequence. The nucleotide identity between these genes is greater than 99.7%. In spite of the low level of divergence between the DNA sequences, the differences in nucleotide sequences reveal one significant non-conserved change in the primary sequence of respective proteins. At position 420, close to ACR5, we found an F in the M2682 strain and an S in the JPCM5 strain (GenBank GIs: JX075891 and 146081774, respectively). We do not have any biochemical data to indicate whether this change could reflect differences in ENTPDase activities of these proteins, but it is reasonable to expect that the ACR5 region has a role in the NTPDase activity in this family [32]. Thus, we believe that the study of this point mutation could be a good focus of future investigations.

**Figure 3:**
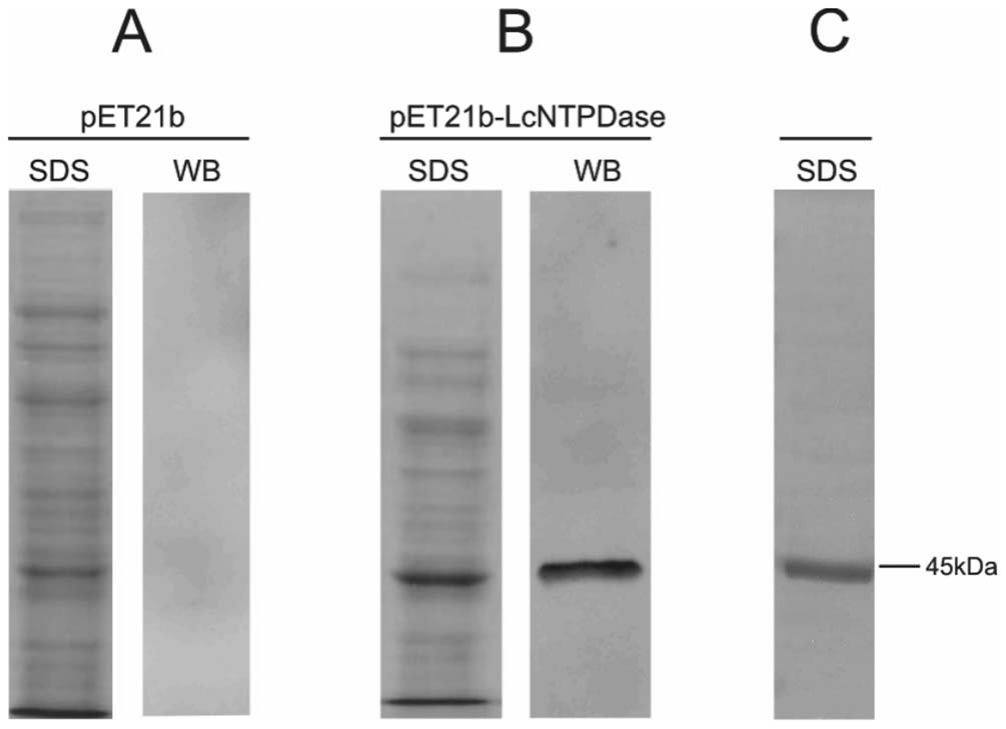
Analyses of expression and purification of rLicNTPDase-2. (A) Protein extract from *E. coli* carrying the empty vector pET21b. (B) Protein extract from *E. coli* carrying the vector pET21b plus rLicNTPDase-2. (C) Purified rLicNTPDase-2 (3 µg) stained by the silver method. SDS lanes indicate samples analyzed after SDS (10%)-PAGE stained with Coomassie blue. WB lanes indicate the same SDS-PAGE samples analyzed by Western blot using anti-His produced in rabbit as primary antibody (1∶4000) and anti-rabbit-IgG conjugated with FITC as secondary antibody (1∶6000). The nitrocellulose membrane was analyzed using an FLA 5100 (Fujifilm) instrument at 475 nm, with a blue filter.

The soluble/ecto domain of rLicNTPDase-2 (L41 to E425) was cloned into the bacterial expression vector pET21b (+). The expression was performed in *E. coli* BL21 (DE3) RIL cells. This construct displayed a satisfactory overexpression of the recombinant protein (Figure 3). The recombinant protein was refolded and purified by affinity chromatography, resulting in a unique band protein with the expected molecular weight of 43.96 kDa as visualized by silver staining (Figure 3C). The expression of rLicNTPDase-2 was confirmed by Western blot (Figure 3B and C).

### Biochemical characterization of rLicNTPDase-2

**Figure 4:**
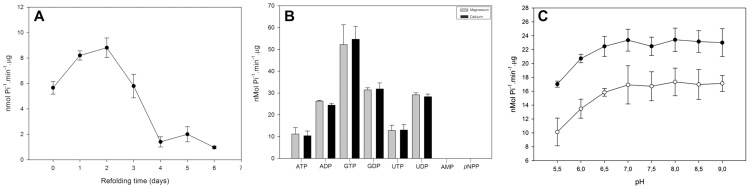
rLic-NTPDase-2: refolding, substrate preference and pH dependence. (A) Refolding assay- the enzymatic activity was measured just after the refolding (zero point) and up to six days later using ADP as the substrate. (B) The preference for different substrates was assessed in the presence of the cofactors calcium (grey bars) or magnesium (black bars). Inset shows the hydrolysis values with SD. (C) ATP (open circles) and ADP (black circles) were used to evaluate enzymatic activity as a function of pH. The pH-dependence test was performed in buffer containing 50 mM MES, 50 mM Tris, 50 mM HEPES, 3 mM MgCl_2_, 116 mm NaCl, 5.4 mM KCl and 2.5 mM nucleotide. The SDs represent the those from the average of three independent experiments performed in triplicate. The free phosphate released was measured using the malachite green method.

Two of the main characteristics of ENTPDases are the ability to use a broad range of nucleotides as substrates and the dependence on divalent cations, mainly calcium or magnesium, as cofactors [33],[34]. The first step of the rLicNTPDase-2 biochemical characterization was analysis of the substrate specificity using calcium or magnesium as co-factors. The rLicNTPDase-2 nucleotidase activity was determined using the substrates ATP, ADP, GTP, GDP, UTP and UDP. To rule out the possibility that the protein preparation contained any phosphatases, an assay performed using p-nitrophenylphosphate (*p*NPP), a substrate of acid and alkaline phosphatases, showed no detectable activity. rLicNTPDase-2 was capable of hydrolyzing all tested triphosphate and diphosphate nucleotides. Under the tested conditions (excess substrate), we observed the highest activity for GTP and the least activity for ATP. The enzyme showed maximum activity between the first and second day after refolding (Figure 4A). Additionally, as shown in Figure 4B, the enzyme did not discriminate between calcium or magnesium ions as cofactors. To assess whether this promiscuity would depend only on a divalent cation regardless of the ion, we tested other divalent cations as cofactors (nickel and zinc), and both abolished the ATPase activity.

Despite the potent rLicNTPDase-2-mediated hydrolysis of GTP, the nucleotides ATP, ADP, UTP and UDP are the most studied due to their important roles in cell signaling processes. We can assume that the hydrolysis of ADP could inhibit the activation of P2Y_1_, P2Y_12_ and P2Y_13_ [35]. These P2 receptors are involved with the production of the inflammatory mediators TNF-α, IL-1 and IL-2 [35]–[37]. Furthermore, hydrolysis of ADP would provide a substrate for the ecto-5'-nucleotidase, which could hydrolyze AMP to adenosine. A2 receptors are activated by adenosine, leading to anti-inflammatory effects such as inhibition of the production of TNF-α, IL-6 and IL-8 [37]. The difference in response to experimental infection with *Leishmania* has been attributed to its ability to hydrolyze nucleotides, resulting in decreased production of interferon-γ and TNF by the lymph nodes and reduced proliferation of spleen cells and germinal centers [10]. Therefore, the mechanism of infection of *L. infantum chagasi* could involve facilitation by regulation of inflammation through NTPDases and another ectonucleotidase.

We next studied the influence of pH on the nucleotidase activity of rLicNTPDase-2. To evaluate the influence of pH on the nucleotide hydrolysis by rLicNTPDase-2, we performed enzymatic activity assays with ATP and ADP under different pH conditions, ranging from 5.5 to 9.0, using magnesium as cofactor (Figure 4C). The enzyme activity was not affected by the variation in pH. This result suggests that this enzyme could be active in different pH environments that occur during the *Leishmania* biological cycle.

### Enzymatic activity assays with apyrase inhibitors

**Figure 5:**
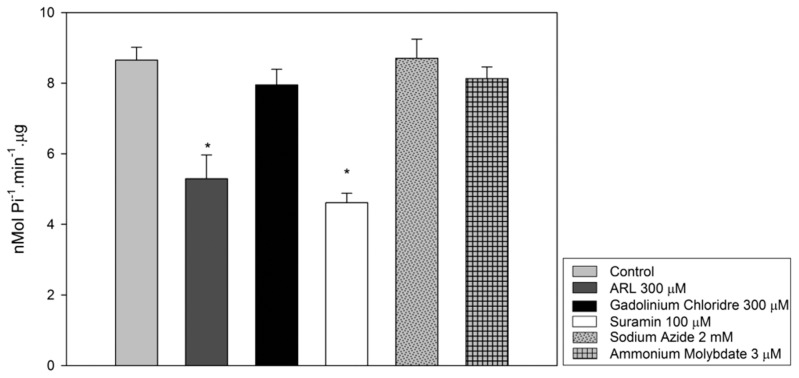
rLicNTPDase-2 ATPase activity in the presence of known partial inhibitors of ENTPDases and a 5′nucleotidase inhibitor. Purified rLicNTPDase-2 was assayed in the absence of inhibitors (control column) or in the presence of inhibitors: ARL 67156 (ARL) 300 µM, gadolinium chloride 300 µM, suramin 100 µM, sodium azide 2 mM and ammonium molybdate 3 µM (a 5′nucleotidase inhibitor). The SDs represent those from the average of three independent experiments performed in triplicate. The free phosphate released was measured using the malachite green method. Statistical analyses were performed using ANOVA, and the significant differences between the control and inhibitors assays are shown with asterisks (p<0.05).

To prove that rLicNTPDase is a genuine apyrase, we tested various partial inhibitors of E-NTPDases described in previous works. We used the following agents: ARL 67156 (6-N, N-Diethyl-bc-dibromomethylene-D-adenosine-5-triphosphate), considered to be a selective inhibitor of ecto-ATPase and able to partially inhibit the NTPDase-1 of *T. cruzi* [8], [38]; gadolinium, a lanthanide capable of inhibiting the ecto-NTPDase of the electric organ of *Torpedo* [39]; suramin, a naphthylurea polysulfone compound that has been shown to be a partial inhibitor of the ecto-ATPDase and the NTPDase-1 of *T. cruzi* [8], [40]; sodium azide, a mitochondrial ATPase inhibitor that is also able to partially inhibit the ecto-ATPase of *T. cruzi* [14] and ammonium molybdate, an inhibitor of 5'-nucleotidase and acid phosphatase [41]. ARL 67156 and suramin partially inhibited the enzyme activity, approximately 38.5% and 46.83%, respectively (Figure 5). No significant inhibition was observed using the other tested compounds. On the other hand, a previous study using live promastigotes from *L. infantum* demonstrated a significant inhibition of ecto-ADPase (80%) and ecto-ATPase activity (24%) by sodium azide [42]. This conflicting result may be explained by an action of azide on ecto-nucleotidases or NTPDases other than the isoform named here as LicNTPDase-2. Another possibility is that under the conditions assayed in live parasites, this isoform could be partially inhibited by azide. Regarding the recombinant enzyme NTPDase-1 of *T. cruzi*, the compound that showed the greatest inhibition was 100 µM suramin (∼50%), while 300 µM gadolinium inhibited only approximately 20% of the activity [8]. These results indicate that despite belonging to the same family and having the five ACR conserved regions, there are differences in the sensitivity of the ENTPDases from different parasites to inhibitors.

Our results shown that rLicNTPDase-2 is truly an apyrase, which has five conserved regions and hydrolyzes tri and diphosphate nucleotides. Under the conditions used, all nucleotides tested were hydrolyzed. Because of the importance of uridine and adenine nucleotides in purinergic signaling and the evidence of the ability of this enzyme to hydrolyze them, we suggest that the enzyme could modulate the host's immune system by decreasing the inflammatory response. Additionally, the NTPDase enzymes have been shown to be important in virulence and replication of trypanosomatids [8], [11], [14], [43]–[45]. In this context, studies of these proteins may help to develop new approaches to therapy. In particular, the study of the crystal structure could contribute to the rational design of new drugs and in better understanding of enzymatic properties of this protein.

### Ecto-nucleotidase activity, expression and localization of rLicNTPDases in *L. infantum chagasi* promastigotes

**Figure 6:**
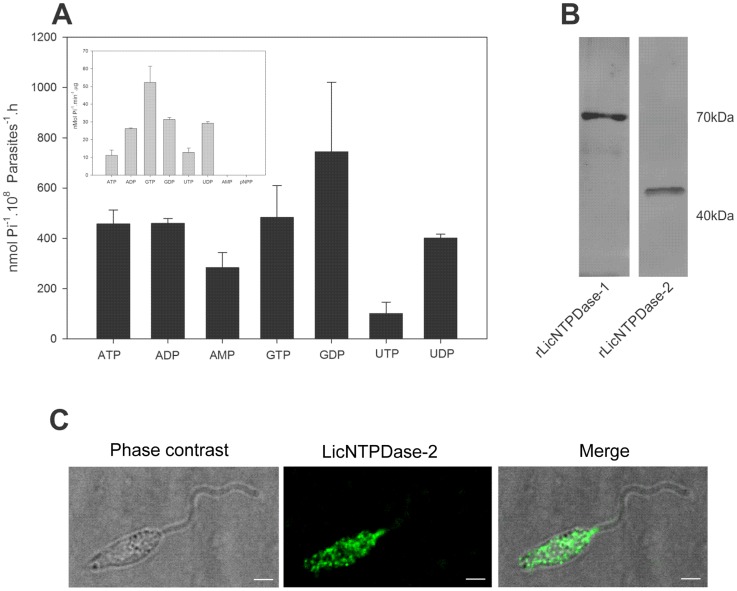
*L. infantum chagasi* promastigote ecto-nucleotidase activity and surface ENTPDase localization using anti-rLicNTPDase-2. (A) The promastigote ecto-nucleotidase activity assays were performed in live total promastigotes from log phase growth using different substrates and Mg^2+^ as cofactor. The activities of recombinant enzyme (rLicNTPDase-2) are shown in the inset box. The SDs represent those of the average of three independent experiments performed in triplicate. The free phosphate released was measured using the malachite green method. (B) Western blotting using anti-rLicNTPDase-2 recognizes both recombinant isoforms rLicNTPDase-1 and rLicNTPDase-2. Purified rLicNTPDase-1 and rLicNTPDase-2 were run in 10% SDS-PAGE and blotted onto a nitrocellulose membrane. The membrane was incubated with purified rabbit polyclonal antibodies to anti-rLicNTPDase-2 (1∶100) as the primary antibody and with anti-rabbit IgG conjugated with FITC (1∶10,000) as the secondary antibody. (C) Distribution of ENTPDases at the surface of *L. infantum chagasi* promastigotes. Non-permeabilized cells fixed with paraformaldehyde were incubated with anti- rLicNTPDase-2 (1∶50) as primary antibody and with Alexa 488-conjugated goat anti-rabbit IgG (1∶400) as secondary antibody. The glass slides were mounted with Prolong Gold Antifade Reagent (Molecular Probes) and examined by confocal microscopy (Leica, SP5). Bar scale  = 2 µm.

To evaluate the presence of ecto-nucleotidase activity in *L. infantum chagasi*, we analyzed general ecto-nucleotidase activities directly on live promastigotes. As shown in Figure 6A, the parasites were able to hydrolyze all tested nucleotides (ATP, ADP, AMP, GTP, GDP, UTP and UDP). The hydrolysis of ATP was similar to ADP, GTP, GDP and UDP. UTPase activity was the lowest ecto-nucleotidase activity observed using this approach. These results indicate that this parasite possesses broad ecto-nucleotidase activity on its surface because no permeabilization was made in this assay. Using another *L. infantum s*train (BH46) and a reaction medium with a different composition, Maia *et al*. [42] found similar results indicating a broad ecto-nucleotidase activity in *L. infantum* promastigotes.

The recombinant enzyme activity was compared with the activity of live promastigotes (Figure 6A-inset). The results indicate a similarity in the broad substrate preference pattern, but differences in the rates of hydrolysis, possibly due to the presence of other ectonucleotidases or of unknown parasite surface environment factors. The hydrolysis of AMP occurs only in the parasite and is due the action of other ectonucleotidases because the NTPDases are unable to hydrolyze monophosphate nucleosides.

E-NTPDases encoded by *Leishmania major, Leishmania infantum, and Leishmania braziliensis* have either a predicted N-terminal transmembrane domain suggesting that they may be anchored on the membrane surface of the parasite or an N-terminal signal peptide to be secreted [45]. Then, to determine the localization of these proteins in the surface of the parasite we performed immunofluorescence in non-permeabilized cells using purified antibody against recombinant E-NTPDase. This antibody recognized both recombinant isoforms rLic NTPDase-1 and rLic NTPDase-2 in Western blotting analysis (Figure 6B) and because of this we cannot distinguish between these isoforms in any immunolocalization assays. Confocal microscopy analysis showed that these proteins are localized to the cell body surface, most concentrate at the anterior end of the cell and no fluorescence were observed in the flagella. This pattern distribution is similar to previously shown for NTPDase in *T. cruzi* [30], in *Leishmania (Viannia) braziliensis* [43] and in *Leishmania* amazonensis [46].

**Figure 7:**
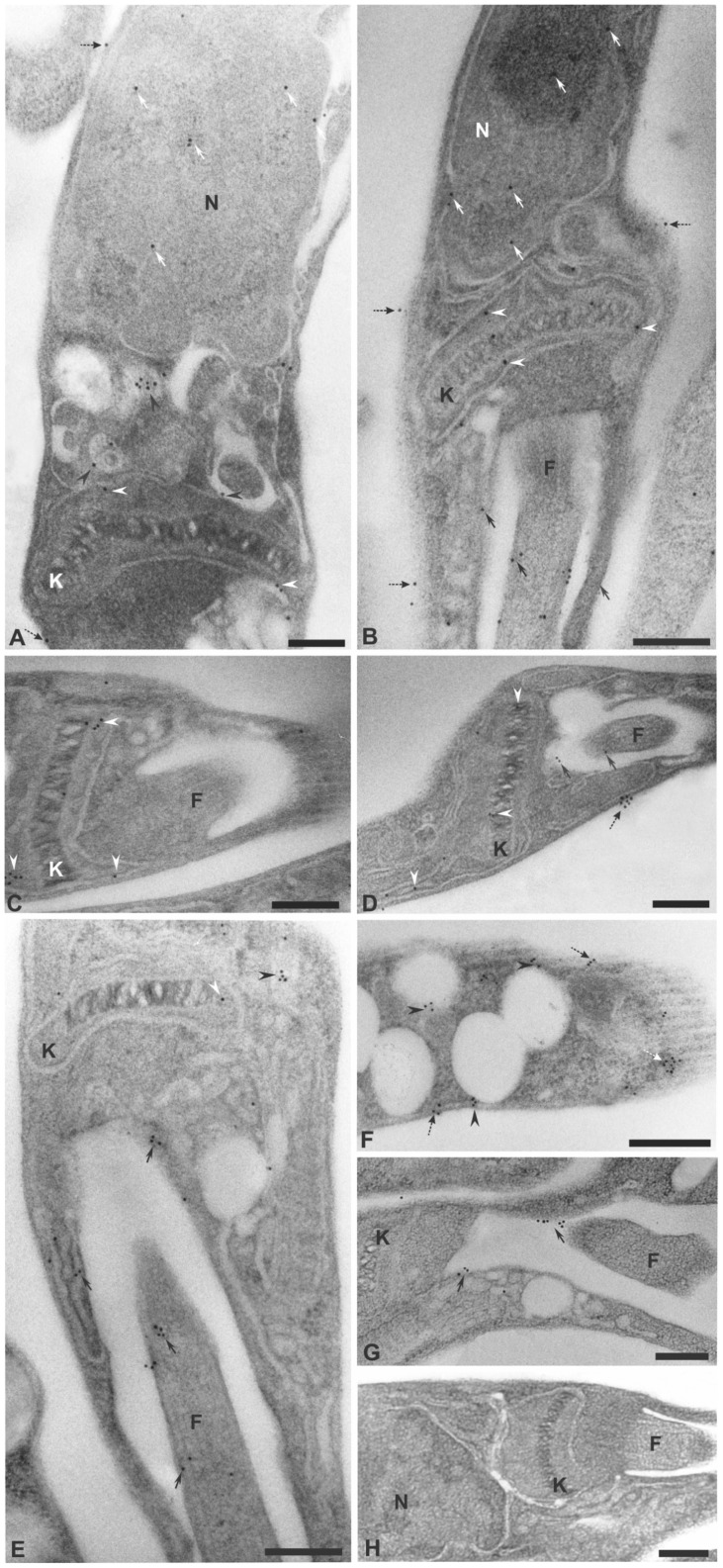
Sub-cellular localization of ENTPDases in *L. infantum chagasi* promastigotes. Electron micrographs using polyclonal antibodies to rLicNTPDase-2 anti-IgG conjugated to 10 nm colloidal gold (A–H). Letters and symbols indicate different localizations: nucleus (N) (white arrow), mitochondria and kinetoplasts (K) (white arrowhead), internal vesicles (black arrowhead), flagellum (F) and flagellar pocket (black arrow) and cell surface (dashed black arrow). No staining was observed in the control (H). Bars: A, C, F, G = 0.1 µm and B = 0.3 µm, C, D and E, H 0.2 µm.

The next step in this work was a detailed investigation of the localization of the TpNTPDases by electron microscopy to confirm the presence of ENTPDases on the cell surface, as observed by the ecto-nucleotidase activity (Figure 6A) and confocal data (Figure 6C). Figure 7A–G shows the presence of the protein on the cell surface, nucleus, flagellum and flagellar pocket region. Additionally, the gold particles stained the kinetoplast, mitochondria and internal vesicles. No staining was observed in the control assay (Figure 7H). The intracellular localization profile is similar to recent studies of *L. braziliensis* and *L.amazonensis* promastigotes [43], [47] and *T. cruzi* [30] epimastigotes and reinforces the ubiquitous localization of these proteins and the requirement for further investigations in this area.

### Recombinant rLicNTPDase2 influence *L. infantum chagasi* adhesion to and infection of macrophages

**Figure 8:**
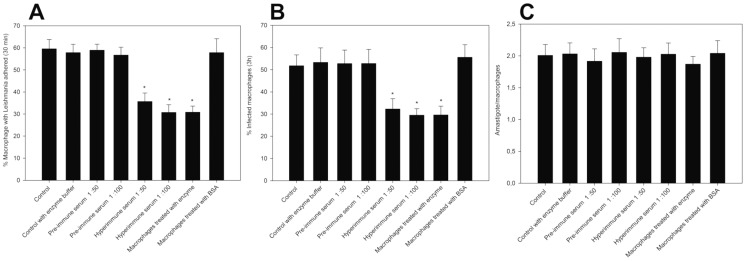
Evaluation of role of LicNTPDases in the infection and adhesion of *L. infantum chagasi* to macrophages. (A) Adhesion of *Leishmania* promastigotes to macrophages. (B) Infection of macrophages by *Leishmania* promastigotes. (C) Treatment did not affect the amount of parasites in macrophages. In all assays (A, B and C): Macrophages treated with polyclonal antiserum anti-rLicNTPDase-2 prior to the infection or with the purified rLicNTPDase-2 before the contact with parasites are compared with macrophages treated with the parasites in the absence of intervention. Control  =  adhesion and infection assay without any intervention. Control with enzyme buffer  =  adhesion and infection assay in the presence of the buffer used to suspend rLicNTPDase-2. BSA was used as a non-related protein. The data reflect the mean + SE from three analyzed slides from each of three independent assays. The asterisks indicate significant differences (*p*<0.05) between the control and other samples.

Previous work demonstrated a possible participation of the trypanosomatid ENTPDases in host infection. Here, we evaluated the influence of rLicNTPDase-2 and the polyclonal antibodies against this recombinant protein in the adhesion and infection of *L. infantum chagasi* in cultured macrophages. The non-related protein bovine serum albumin (BSA) and pre-immune serum were used as negative controls. When the macrophages were treated with the recombinant enzyme before the addition of parasites, there was a 48.3% reduction of adhesion and 43.91% reduction of infection (Figure 8 A–B). However, parasite proliferation (Figure 8C) was not influenced by any treatments, demonstrating that the effect observed in the adhesion step does not seem to influence the viability of internalized parasites. Taken together, these results suggest the possible existence of binding sites (e.g., receptors) for rLicNTPDase2 in the macrophages that may facilitate adherence and infection. Specifically, by binding to these receptors, the recombinant enzyme would most likely prevent the interaction of the parasite enzyme with the macrophage (Figure 8). However, we cannot exclude other possible explanations as discussed by Mariotini-Moura and co-workers [30]. Anti-rLicNTPDase-2 was also able to significantly reduce the adhesion (48.41% at 1∶100 dilution and 40.1% at 1∶50 dilution of the hyperimmune serum) and infection (45.4% at 1∶100 and 37.7% at 1∶50). We can speculate that the antibodies would bind to the parasite rLicNTPDases 1 and/or 2, preventing its interaction with a putative macrophage receptor. However, we cannot ignore other hypothesis such as the presence of unknown molecules that could interfere with the binding of rLicNTPDase to the host cells. Regardless of the mechanism, this area needs to be further investigated.

### rLicNTPDases are expressed in naturally infected dogs

**Figure 9:**
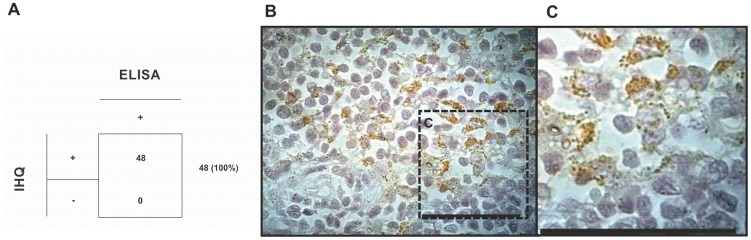
Immunohistochemistry using anti-rLicNTPDase-2 in the lymph nodes of naturally infected dogs. (A) Lymph nodes from 48 *Leishmania*-positive dogs were evaluated by immunohistochemistry (IHC) using anti-rLicNTPDase-2. The results of the IHC are compared with ELISA data of the same samples using a Biomanguinhos Kit. (B) An example of IHC result using polyclonal anti-rLicNTPDase-2. (C) The zoom of section C is from image B.

In our previous work, we demonstrated that the recombinant Lic-NTPDase-2 is a good novel antigen for immunological diagnosis of canine visceral leishmaniasis [21]. That work suggested the presence of active *Leishmania* in dogs but only via an indirect method because we used the recombinant protein as a target to measure the levels of specific antibodies in samples of serum from dogs. Here, we directly investigated the presence of amastigotes in naturally infected dogs. *Leishmania* can infect many different organs of mammalian hosts. In this work, we used immunohistochemistry with antibodies against rLic-NTPDase-2 to directly evaluate the expression of LicNTPDases in the lymph nodes of naturally infected dogs (Figure 9). In this approach, 45 samples (95.7%) showed immunoperoxidase staining, directly demonstrating the presence of the recognized antigen in the tissue. These data show for the first time the expression of LicNTPDases in tissues from naturally infected dogs and corroborate previous data from our group that demonstrated the potential application of rLicNTPDase-2 in the diagnosis of canine Leishmaniasis [21].

### Conclusions

In this work we expanded the study of ENTPDases from *L. infantum chagasi* (syn. *L. infantum*). The bioinformatics studies shown the presence of two ENTPDase paralogs (named here as NTPDase-1 and NTPDase-2) in kinetoplastids, except for *T. cruzi*, in which only one member of this family (the known NTPDase-1 described previously) was observed [14]. In the context of the low levels of identity with mammalian ENTPDases and the previous knowledge of the *T. cruzi* NTPDase-1, we propose here a new nomenclature for the trypanosomatid ENTPDases: TpNTPDase-1 for the trypanosomatid ENTPDases more similar to *T. cruzi* E-NTPDase-1 (∼70 kDa proteins) and TpNTPDase-2 for the lower molecular weight isoform (∼40 kDa), which is absent from *T. cruzi*.

In addition, a new search for TpNTPDases in the molecular protein databanks demonstrated that trypanosomatides, in general, have the two isoforms of TpNTPDases and that *T. cruzi* remains as a unique trypanosomatide that possesses only the TpNTPDase-1 isoform.

The biochemical characterization of recombinant Lic-NTPDase-2 shows that it is a genuine nucleotidase/apyrase from the CD39/GDA1 family because the enzyme hydrolyzes a broad spectrum of tri and diphosphate (but not monophosphate) nucleotides, is dependent on divalent cations (Mg^++^ or Ca^++^) and is partially inhibited by known CD39 family partial inhibitors. In addition, we showed that live *L. infantum chagasi* promastigotes have ecto-nucleotidase activity, and the immunolocalization shows the expected ENTPDases on the surface of promastigotes. However, our results also indicated a broad intracellular expression of the ENTPDases, opening new fields of investigation into other previously unknown biological roles. This point is interesting because we observed the presence of these enzymes in unexpected localizations such as the kinetoplastids, mitochondria and nuclei.

In addition we investigated the role of rLicNTPDase-2 during the adhesion to and infection of host cells, and our results demonstrated that this protein participates in these processes, most likely acting as a facilitator of infection as previously observed for *T. cruzi* infection [9], [30]. Furthermore, the importance of ENTPDases in natural infection was indicated by direct detection of their expression in tissues from naturally infected dogs. Taken together, these results reinforce the application of rLicNTPDase-2 in the diagnosis of visceral leishmaniasis as previously described [21] and may suggest that the ENTPDases could be used in other biotechnological applications including drug and vaccine development. Both applications are under investigation in our research group.

The NTPDase enzymes have been shown to be important in virulence and replication of trypanosomatids [8], [11], [14], [43]–[45]. In this context, research on these proteins may help to develop new approaches to therapy for this disease. In particular, the elucidation of the crystal structure could contribute to the rational design of new drugs and better understanding of how these enzymes participate in the host-pathogen interaction.

## Supporting Information

Figure S1 **Alignment of the ENTPDases from mammals and Trypanosomatids (** ***Leishmania*** **and** ***T. cruzi*** **).** ENTPDase sequences were aligned by the CLC workbench program. The alignment was manually inspected guided by the ACRs pairing. The two putative proteins from *L. infantum* genome were aligned with those of *Leishmania*, *Trypanosoma cruzi* and the mammalian ENTPDases representative of isoforms 1 to 8 from human and mouse. GeneBank accession numbers: NTPDase1-Hs_gi|1842120|, NTPDase1-Mm_gi|6753346|, NTPDase2-Hs_gi|5114239|, NTPDase2-Mm_gi|36312789|, NTPDase3-Hs_gi|13817037|, NTPDase3-Mm_gi|36312771|, NTPDase4-_Hs_gi|3153211|, NTPDase4-Mm_gi|18093090|, NTPDase5-Hs_gi|3335102|, NTPDase5-Mm_gi|5139519|, NTPDase6-Hs_gi|32966069|, NTPDase6-Mm_gi|83921570|, NTPDase7-Hs_gi|9623384|, NTPDase7-Mm_gi|9858131|, NTPDase8-Hs_gi|37813200|, NTPDase8-Mm_gi|35293542|, L_major45kDa XP_001681917, L_infantum45 kDa L_braziliensis45 kDa XP_001562178, L_major70 kDa XP_001681345, L_infantum70 kDa XP_001463665, L_braziliensis70 kDa gi|XP_001562788, Tcruzi70 kDa_gi|45685733|. *Mus musculus* (Mm); *Homo sapiens* (Hs).
(TIF)

Figure S2 **Phylogenetic analysis performed by Bayesian inference based in 29 amino acid sequences of Trypanosomatides NTPDases (TpNTPDase) proteins.** The phylogenetic tree was constructed using the Bayesian inference (BI) method with the software MrBayes v3.1.2. The values of posteriori probability (PP) were calculated using the best tree, and are expressed in percentages beside of each node. *Bootstraping* values (BV) are also expressed in percentages below of some node. The BV represent the percentage of trees in which the associated species clustered together in the tree produced by Maximum likelihood method. Species names are represented by the first letter of genus, capitalized, (L to *Leishmania* and T to *Trypanosoma*) followed by name of the species. Strains are underlined and the sequences IDs in bold correspond to the sequences extracted either from the NCBI or TriTrypDB. The outgroup taxon is *Toxoplasma gondii*.
(TIF)
