# Supplementary figures and images for: Leishmania infantum Ecto-Nucleoside Triphosphate Diphosphohydrolase-2 is an Apyrase Involved in Macrophage Infection and Expressed in Infected Dogs
Source: PLoS Negl Trop Dis. 2014 Nov 13;8(11):e3309. doi: 10.1371/journal.pntd.0003309 (PMC4230930; doi:10.1371/journal.pntd.0003309)

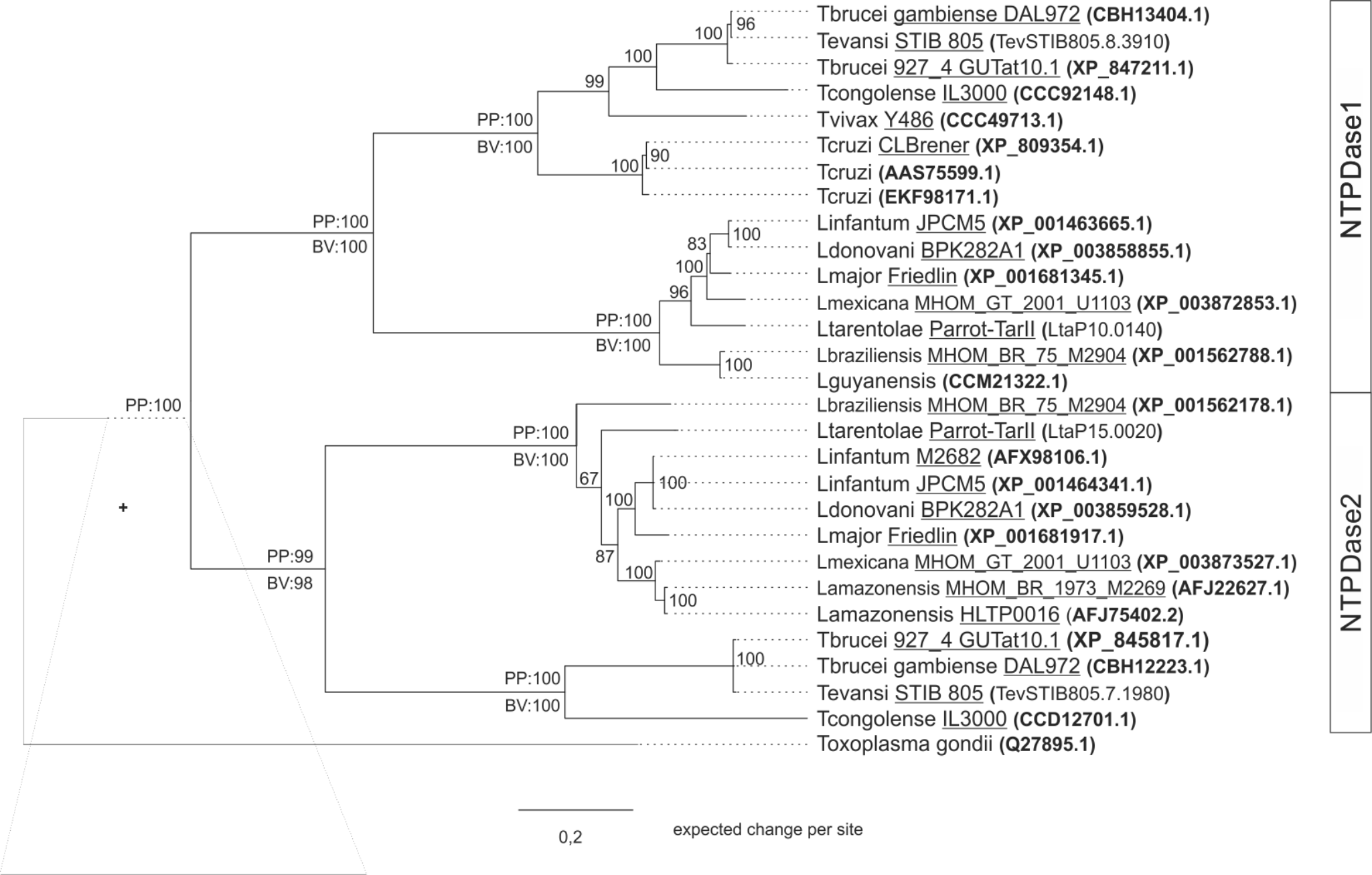

Supplement: Figure S2 — Phylogenetic analysis performed by Bayesian inference based in 29 amino acid sequences of Trypanosomatides NTPDases (TpNTPDase) proteins. The phylogenetic tree was constructed using the Bayesian inference (BI) method with the software MrBayes v3.1.2. The values of posteriori probability (PP) were calculated using the best tree, and are expressed in percentages beside of each node. Bootstraping values (BV) are also expressed in percentages below of some node. The BV represent the percentage of trees in which the associated species clustered together in the tree produced by Maximum likelihood method. Species names are represented by the first letter of genus, capitalized, (L to Leishmania and T to Trypanosoma) followed by name of the species. Strains are underlined and the sequences IDs in bold correspond to the sequences extracted either from the NCBI or TriTrypDB. The outgroup taxon is Toxoplasma gondii. (TIF) [file pntd.0003309.s002.tif]
